# Supplementary material for: TPL‐2 kinase induces phagosome acidification to promote macrophage killing of bacteria
Source: EMBO J. 2021 Apr 21;40(10):e106188. doi: 10.15252/embj.2020106188 (PMC8126920; doi:10.15252/embj.2020106188)
Supplement: Supplementary file 1 — Expanded View Figures PDF [file EMBJ-40-e106188-s002.pdf]

Expanded View Figures

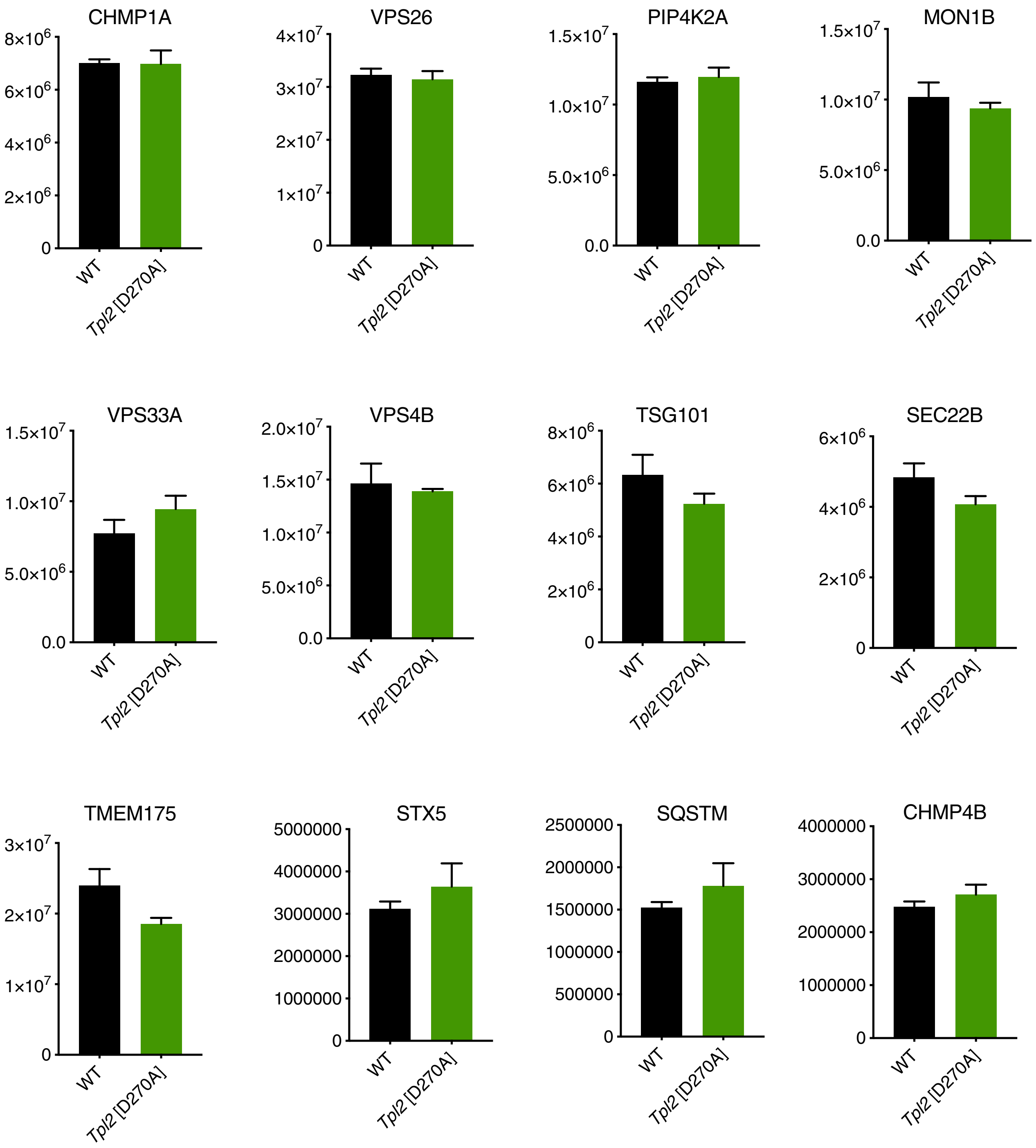

Figure EV1. *Tpl2*[D270A] mutation does not alter abundance of several key phagosomal proteins on isolated phagosomes.

Protein intensities of selected proteins from phagosomes purified from WT and *Tpl2*[D270A] BMDMs (n = 3 biological replicates). Error bars represent SEM.

 © 2021 The Authors

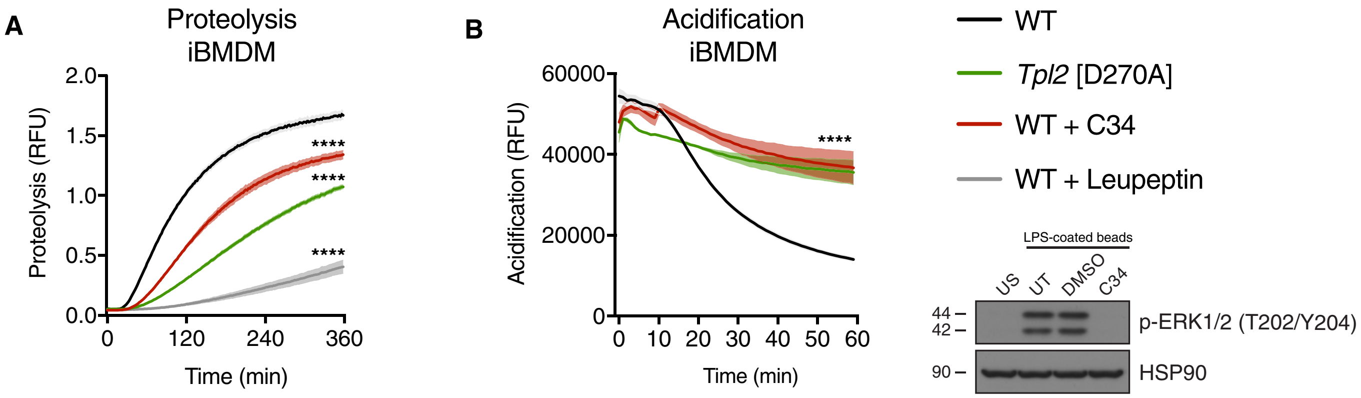

Figure EV2. TPL-2 kinase activity promotes phagosomal proteolysis and acidification in iBMDMs.

A Intra-phagosomal proteolysis of DQ Green BSA / AF594 latex beads in iBMDMs pre-treated with 10 μM C34 TPL-2 inhibitor for 1 h (*n* = 4 wells). WT cells were pre-treated with leupeptin to block proteolysis.

B Intra-phagosomal acidification of BCECF-coupled latex beads in iBMDMs pre-treated with 10 μM C34 TPL-2 inhibitor for 1 h (*n* = 4 wells).

Data information: One representative experiment out of three shown. Shaded areas represent SEM. ****$P < 0.0001$. Paired Mann–Whitney *t*-test. All differences relative to WT are ****.

Figure EV3. TPL-2 regulates phagosome proteolysis via DMXLI.

A *Dmxl1* was knocked down in WT iBMDMs by RNA interference using a SMARTpool ON-TARGETplus siRNA for 48 h. ON-TARGETplus non-targeting pool functioned as siRNA control. Intra-phagosomal proteolysis was assayed following uptake of DQ Green BSA / AF594 latex beads (*n* = 4 wells).

B–D *Dmxl2* was knocked down in WT iBMDMs by RNA interference using a SMARTpool ON-TARGETplus siRNA for 48 h. ON-TARGETplus non-targeting pool functioned as siRNA control. (B) Intra-phagosomal proteolysis was assayed following uptake of DQ Green BSA / AF594 latex beads (*n* = 4 wells). (C) Intra-phagosomal acidification was assayed following uptake of BCECF-coupled latex beads (*n* = 4 wells). (D) qRT–PCR analysis of RNA extracted from iBMDMs was used to check the efficiency of *Dmxl2* knockdown (A and B). *Dmxl2* mRNA levels were normalised to *Hprt* mRNA levels and fold changes calculated (ΔC_t values) (*n* = 4).

E *Dmxl1* was knocked down in *Tpl2*[D270A] iBMDMs by RNA interference using a SMARTpool ON-TARGETplus siRNA for 48 h. ON-TARGETplus non-targeting pool functioned as siRNA control. Intra-phagosomal proteolysis was assayed following uptake of DQ Green BSA / AF594 latex beads (*n* = 4 wells).

F Simultaneous with *Dmxl1* siRNA knockdown, WT iBMDMs were co-transfected with plasmids expressing either 3xFLAG-DMXL1 (1,773–2,047) or 3xFLAG-DMXL1 S1903A/S1904A (1,773–2,047). Intra-phagosomal proteolysis was assayed following uptake of DQ Green BSA / AF594 latex beads (*n* = 4 wells).

G Simultaneous with *Dmxl1* siRNA knockdown, *Tpl2*[D270A] iBMDMs were co-transfected with a plasmid expressing 3xFLAG-DMXL1 (1,773–2,047). Intra-phagosomal proteolysis was monitored (*n* = 4 wells).

Data information: (A-G) One representative experiment out of three shown. Error bars and shaded areas represent SEM. ****$P < 0.0001$. Paired Mann–Whitney *t*-test. All differences relative to WT are ****. UT, untransfected; NT, non-targeting siRNA pool.

© 2021 The Authors

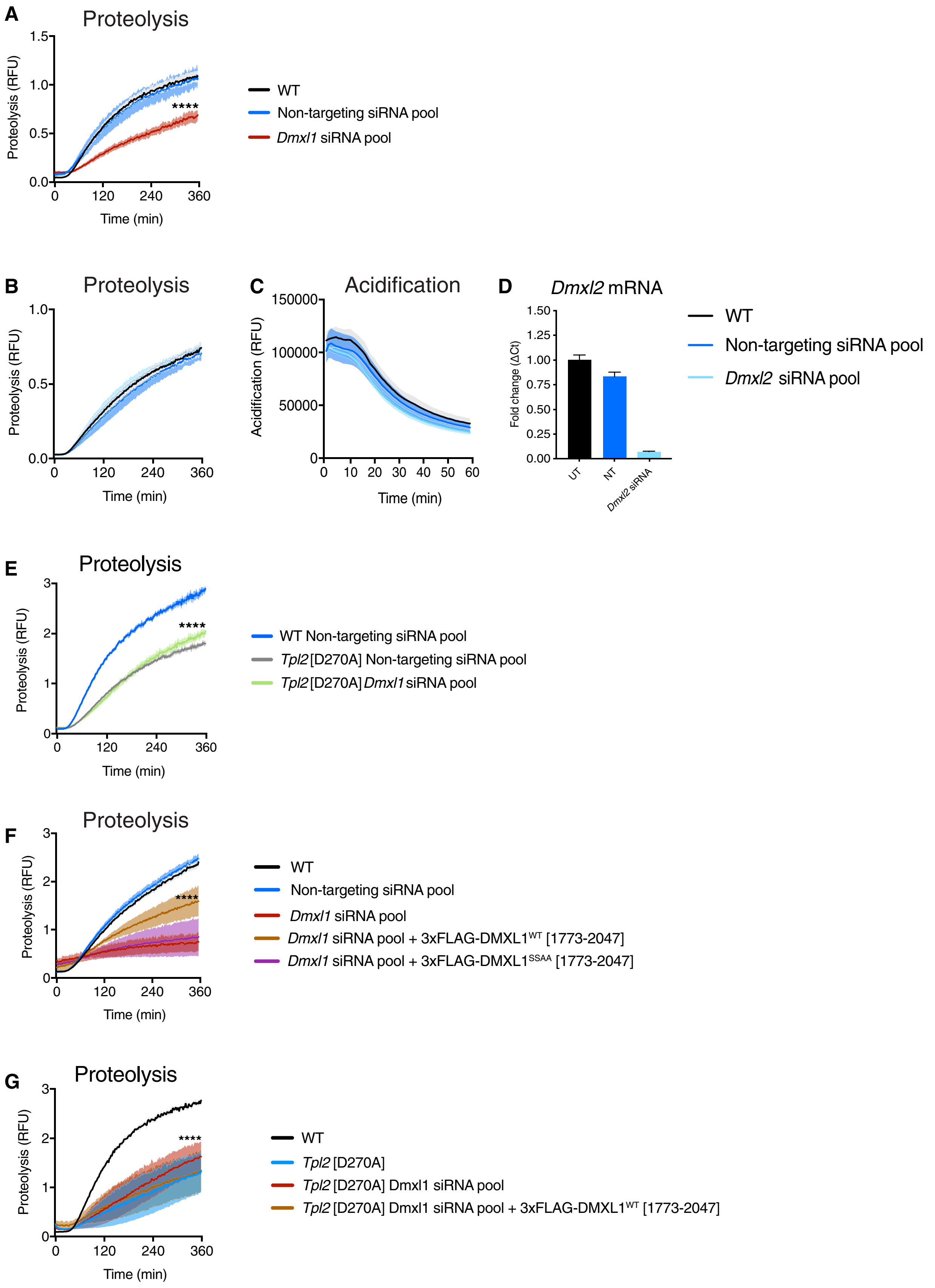

Figure EV3.

 © 2021 The Authors
